# Ophthalmic artery Doppler as potential surrogate marker of angiogenic imbalance in near‐term pregnancy

**DOI:** 10.1002/uog.70270

**Published:** 2026-06-28

**Authors:** M. F. Tinajero, C. Faraci, M. Cuenca, M. Ferrer, S. Paolucci, E. Gratacos, F. Crovetto, F. Figueras

**Affiliations:** ^1^ BCNatal, Hospital Clínic and Hospital Sant Joan de Déu Barcelona Spain; ^2^ Facultat de Medicina i Ciències de la Salut Universitat de Barcelona Barcelona Spain; ^3^ Department of Obstetrics and Gynaecology Università di Brescia, Ospedali Civili di Brescia Brescia Italy; ^4^ Department of Women and Child Health Women Health Area, Fondazione Policlinico Universitario Agostino Gemelli IRCCS Rome Italy; ^5^ Institut d'Investigacions Biomèdiques August Pi I Sunyer (IDIBAPS) Barcelona Spain; ^6^ Centre for Biomedical Research on Rare Diseases (CIBER‐ER) Madrid Spain; ^7^ Institut de Recerca Sant Joan de Déu (IRSJD) Barcelona Spain; ^8^ Spanish Network in Maternal, Neonatal, Child and Developmental Health Research, RICORS‐SAMID, RD24/0013/0004, Instituto de Salud Carlos III Madrid Spain

**Keywords:** angiogenic factor, Doppler ultrasonography, ophthalmic artery, placental growth factor, pre‐eclampsia, soluble fms‐like tyrosine kinase‐1, uterine artery

## Abstract

**Objectives:**

First, to evaluate whether the ratio of soluble fms‐like tyrosine kinase‐1 to placental growth factor (sFlt‐1/PlGF) is associated with hemodynamic changes in ophthalmic artery (OA) Doppler in near‐term pregnancy. Second, to assess the performance of OA Doppler to rule in and rule out angiogenic factor imbalance.

**Methods:**

This was a cross‐sectional cohort study nested within the PE37 randomized controlled trial, involving nulliparous women recruited between January 2023 and January 2025 who underwent sFlt‐1/PlGF ratio measurement between 35 + 0 and 36 + 6 weeks' gestation. We included a subsample of women who underwent OA Doppler evaluation, including measurement of OA peak systolic velocity (PSV) ratio and OA pulsatility index (PI), as well as assessment of mean arterial pressure (MAP) and mean uterine artery (UtA) PI. The operator was blinded to sFlt‐1/PlGF ratio values. Trends in median values of OA and maternal–fetal Doppler parameters across sFlt‐1/PlGF tertiles were analyzed using the Jonckheere–Terpstra test and quantile regression, adjusting for maternal body mass index, age and smoking status. The predictive performance of the OA‐PSV ratio for sFlt‐1/PlGF ratio ≥ 38 was evaluated using receiver‐operating‐characteristics‐curve analysis.

**Results:**

We included 203 women, of whom 62, 71 and 70 were in the lowest, middle and highest tertiles of the sFlt‐1/PlGF ratio, respectively. With increasing sFlt‐1/PlGF ratio tertile, there was a significant increase in the OA‐PSV ratio (median, 0.45 (interquartile range (IQR), 0.39–0.53) *vs* 0.48 (IQR, 0.41–0.58) *vs* 0.59 (IQR, 0.50–0.66); adjusted *P <* 0.001), a significant decrease in OA‐PI (median, 2.20 (IQR, 1.92–2.61) *vs* 2.13 (IQR, 1.86–2.37) *vs* 1.86 (IQR, 1.60–2.26); adjusted *P* = 0.031) and a significant increase in MAP (median, 87.0 (IQR, 82.7–92.0) mmHg *vs* 88.7 (IQR, 83.7–93.7) mmHg *vs* 94.7 (IQR, 89.3–100.0) mmHg; adjusted *P* < 0.001). In contrast, no significant trend was observed in mean UtA‐PI across sFlt‐1/PlGF ratio tertiles. Among those individuals with an OA‐PSV ratio < 0.61, 90.5% truly had a sFlt‐1/PlGF ratio < 38, at a 15% false‐positive rate.

**Conclusions:**

This study provides new evidence of a significant association between the sFlt‐1/PlGF ratio and OA Doppler parameters in near‐term pregnancies, suggesting that OA Doppler indices, particularly the PSV ratio, reflect angiogenic imbalance. Given its non‐invasive nature, accessibility and low cost, OA Doppler emerges as a promising surrogate tool for ruling out angiogenic imbalance. © 2026 The Author(s). *Ultrasound in Obstetrics & Gynecology* published by John Wiley & Sons Ltd on behalf of International Society of Ultrasound in Obstetrics and Gynecology.

## INTRODUCTION

Pre‐eclampsia affects around 5% of pregnancies worldwide and remains a leading cause of maternal mortality and morbidity^1^. It is also associated with long‐term multisystem sequelae for both mother and offspring^2^, ^3^, ^4^. While preterm pre‐eclampsia is associated strongly with placental insufficiency^5^, term pre‐eclampsia (≥ 37 weeks) is characterized by lower placental involvement, suggesting a greater role for maternal cardiovascular maladaptation^6^ and endothelial dysfunction^7^ in late‐onset disease.

Angiogenic factors have been proposed as biochemical markers of placental and endothelial function^8^. In pre‐eclampsia, an imbalance characterized by decreased placental growth factor (PlGF) and elevated soluble fms‐like tyrosine kinase‐1 (sFlt‐1) is linked to widespread endothelial dysfunction, increased systemic vascular resistance, hypertension and proteinuria^9^, ^10^.

Recognition of the role of angiogenic factors in pre‐eclampsia has led to significant advances in obstetric practice^11^, ^12^. Since endothelial dysfunction precedes disease onset^13^, the sFlt‐1/PlGF ratio is a reliable tool for ruling out pre‐eclampsia^14^. However, the relatively high cost of testing and limited accessibility in low‐resource settings have prompted evaluation of other non‐invasive tests as complementary or alternative tools for predicting endothelial damage and pre‐eclampsia. Among these, ophthalmic artery (OA) Doppler has gained attention^15^.

Growing evidence supports OA Doppler as a promising marker for pre‐eclampsia^15^, ^16^, ^17^, ^18^, ^19^, ^20^. Endothelial damage induced by antiangiogenic molecules may be reflected in OA Doppler waveforms^21^, ^22^. The dicrotic wave characteristic of OA Doppler provides indirect insight into endothelial function by indicating peripheral vascular status^23^. A decrease in OA pulsatility index (PI) and an increase in the ratio of the second (PSV2) to the first (PSV1) peak systolic velocity have been observed under pathological conditions^21^, ^24^, ^25^, ^26^, ^27^.

OA Doppler is easy to perform, reproducible and stable throughout gestation, with low interobserver variability, and it has the capacity to distinguish pre‐eclampsia from other hypertensive disorders^28^, ^29^, ^30^, ^31^. Recently, Lau *et al*.^32^ suggested that OA Doppler could serve as an alternative to the sFlt‐1/PlGF ratio for predicting imminent pre‐eclampsia, when combined with maternal risk assessment and blood pressure measurement. However, evidence of a direct association between OA Doppler indices and the sFlt‐1/PlGF ratio is lacking. Clarifying this relationship could elucidate whether OA Doppler could serve as a surrogate for the sFlt‐1/PlGF ratio.

This study aimed to evaluate whether the sFlt‐1/PlGF ratio is associated with hemodynamic changes in OA Doppler in near‐term pregnancy and, secondarily, to assess the performance of OA Doppler in ruling in and ruling out angiogenic factor imbalance.

## METHODS

### Study design and population

This was a cross‐sectional cohort study of women recruited for the PE37 trial between January 2023 and January 2025 at Hospital Clinic Barcelona, Barcelona, Spain. The PE37 study is an ongoing randomized controlled trial (ClinicalTrials.gov reference: NCT04766866) aimed at reducing the incidence of term pre‐eclampsia using planned delivery for women at high risk, as determined by the sFlt‐1/PlGF ratio at 35 + 0 to 36 + 6 weeks' gestation^33^. The inclusion criteria of the study were nulliparous women over 18 years old with a singleton pregnancy between 35 + 0 and 36 + 6 weeks' gestation, without suspicion of fetal growth restriction or pre‐eclampsia. The present study included a subsample of women recruited for the PE37 trial who underwent OA Doppler evaluation. Written informed consent was obtained from all participants, and the study was approved by the hospital's ethics committee (reference: HCB/2020/1067).

### Measurements

#### Angiogenic factors

Between 35 + 0 and 36 + 6 weeks, serum concentrations of PlGF and sFlt‐1 were measured using automated electrochemiluminescence immunoassays on the Roche Cobas® e 411 analyzer (Roche Diagnostics GmbH, Mannheim, Germany). To evaluate the association between angiogenic factors and OA Doppler parameters, participants were classified into three equally sized groups based on the sFlt‐1/PlGF ratio, adjusted for gestational age, corresponding to the lowest, middle and highest tertiles. The tertile cut‐offs for sFlt‐1/PlGF ratio had been established previously based on a sample of 654 women included in the PE37 study using quantile regression (Table S1). For the secondary analysis, angiogenic imbalance was defined as a sFlt‐1/PlGF ratio ≥ 38^33^.

#### Ophthalmic artery Doppler

OA Doppler was performed using a Voluson P8 ultrasound system (GE Healthcare, Zipf, Austria), equipped with a 12L‐RS linear transducer (field‐of‐view, 37 mm; bandwidth, 4–12 MHz). Pregnant individuals were examined in a semirecumbent position with the back angled at 30–45°. The transducer was placed over the upper eyelid. The optic nerve was identified as a hypoechogenic band, then color Doppler was used to localize the OA, which usually crosses from the temporal to the nasal region. An insonation angle of < 20° was maintained to ensure accuracy. Settings included a 2‐mm sample gate, a depth of 3.0–4.5 cm, a low wall filter (50–60 Hz) and a low pulse repetition frequency set at 4.4 kHz, with manual adjustments between 3 kHz and 6 kHz when necessary to minimize artifacts and optimize the Doppler spectrum. The following Doppler parameters were obtained: OA‐PI, OA‐PSV1, OA‐PSV2 and the OA‐PSV ratio (OA‐PSV2/OA‐PSV1). The machine automatically obtained OA‐PSV1 and OA‐PI, while OA‐PSV2 and the OA‐PSV ratio were measured manually. A single trained operator (M.F.T.) performed all OA Doppler measurements and remained blinded to the sFlt‐1/PlGF ratio results. To ensure consistency and reliability, the following quality criteria were defined: (1) a measurement available in both eyes; (2) OA‐PSV1 > 20 cm/s; and (3) an intereye difference in the OA‐PSV ratio of < 33%^34^.

#### Mean arterial pressure

Maternal blood pressure was measured in a quiet, temperature‐controlled room after 30 min of rest. Three consecutive readings were recorded 1 min apart using the same device and the average was calculated. Mean arterial pressure (MAP) was calculated using the standard formula: diastolic blood pressure (DBP) + one‐third of the difference between systolic blood pressure (SBP) and DBP (MAP = DBP + 1/3 × (SBP – DBP)).

#### Obstetric ultrasound and Doppler

After angiogenic factors had been measured, an obstetric ultrasound scan was performed between 35 + 0 and 37 + 6 weeks to calculate estimated fetal weight (EFW) using Hadlock's formula, based on biparietal diameter, head circumference, abdominal circumference and femur length^35^. The fetal growth centile was calculated according to EFW, sex and gestational age, using local standards^36^. Fetal and uterine artery (UtA) Doppler studies were performed according to the guidelines of the International Society of Ultrasound in Obstetrics and Gynecology (ISUOG)^37^. PI values for the middle cerebral artery and umbilical artery were obtained, and the cerebroplacental ratio was calculated.

#### Other measurements

The following variables were also collected: maternal weight and height measured at the time of evaluation; self‐reported ethnicity; educational level, categorized as low (primary/secondary) or high (university/higher education); current employment status (employed or unemployed); use of assisted reproductive technology; pre‐existing medical conditions; medical conditions during pregnancy; current medication; and smoking before and/or during pregnancy.

### Outcomes

The primary outcomes of this study were the OA‐PSV ratio and OA‐PI, assessed across tertiles of the sFlt‐1/PlGF ratio, adjusted for gestational age. Secondary outcomes included MAP and mean UtA‐PI assessed across the same sFlt‐1/PlGF tertiles.

### Sample size

Based on published data from pregnant individuals during the third trimester, a mean ± SD value of 0.542 ± 0.097 was expected for the OA‐PSV ratio^31^. To perform pairwise comparisons between sFlt‐1/PlGF ratio tertile groups using the independent‐samples *t*‐test, assuming a 5% alpha error, 90% power and a 10% difference in means between groups, a sample size of 69 patients per tertile group was calculated (total *n* = 207).

### Statistical analysis

The normality of data distribution was evaluated using the Shapiro–Wilk test. Continuous variables were compared using one‐way ANOVA. Categorical variables were compared using the chi‐square test or Fisher–Freeman–Halton exact test, as appropriate. Trends across sFlt‐1/PlGF tertiles were assessed using the Jonckheere–Terpstra test. Multivariable associations between Doppler indices and the sFlt‐1/PlGF ratio were analyzed using quantile regression models (treating sFlt‐1/PlGF as an ordinal categorical variable), adjusted for maternal body mass index (BMI), age and smoking status. Pairwise differences between tertile groups were derived from these quantile regression models. A prespecified sensitivity analysis was performed including only cases that met the predefined quality criteria for OA Doppler assessment. Given the number of outcomes evaluated, these analyses were considered exploratory, and no adjustment for multiple testing was applied.

In a secondary exploratory analysis, we evaluated the ability of the OA‐PSV ratio to discriminate a sFlt‐1/PlGF ratio of ≥ 38 using receiver‐operating‐characteristics (ROC)‐curve analysis. Pairwise comparison between ROC curves was performed using DeLong's method^38^.

Statistical analysis was conducted using STATA/BE version 17.0 (StataCorp. LLC, College Station, TX, USA), and graphical outputs were generated using the ggplot2 package in R (R Foundation for Statistical Computing, Vienna, Austria). Statistical significance was set at *P* < 0.05.

## RESULTS

A total of 206 nulliparous pregnant individuals were included and categorized into tertiles based on the value of the sFlt‐1/PlGF ratio measured at 35 + 0 to 36 + 6 weeks: lowest tertile (*n* = 62), middle tertile (*n* = 73) and highest tertile (*n* = 71). Gestational‐age‐specific cut‐off values for the sFlt‐1/PlGF ratio are presented in Table S1. Three participants had incomplete data owing to loss to follow‐up after recruitment, resulting in a sample size of 203 individuals for the primary analysis, of whom 62, 71 and 70 women were in the lowest, middle and highest tertiles of sFlt‐1/PlGF ratio, respectively. Among these 203 participants, 189 met the quality criteria for OA Doppler assessment and were included in the sensitivity analysis.

The baseline characteristics of the study population are detailed in Table 1. Gestational age at the time of blood sampling and Doppler evaluation did not differ between groups. Most participants were White and had a high level of education. No significant differences among tertiles were observed in maternal BMI at presentation, smoking status, pre‐existing medical conditions or mode of conception.

**Table 1:** Demographic and clinical characteristics of 203 women, according to tertile of soluble fms‐like tyrosine kinase‐1 to placental growth factor (sFlt‐1/PlGF) ratio

	sFlt‐1/PlGF ratio tertile			
Characteristic	Lowest (*n* = 62)	Middle (*n* = 71)	Highest (*n* = 70)	*P*
Maternal age (years)	33 ± 5	34 ± 5	35 ± 5	0.064*
BMI at presentation (kg/m^2^)	23.5 ± 3.5	24.0 ± 5.4	24.7 ± 4.8	0.330*
GA at blood sampling (weeks)	36 + 0 ± 2 + 5	35 + 6 ± 2 + 4	35 + 6 ± 2 + 1	0.419*
GA at Doppler assessment (weeks)	36 + 6 ± 3 + 5	36 + 6 ± 3 + 4	37 + 0 ± 3 + 2	0.519*
Self‐reported ethnicity				0.350†
White	42 (67.7)	48 (67.6)	42 (60.0)	
Hispanic	19 (30.6)	19 (26.8)	21 (30.0)	
Other	1 (1.6)	4 (5.6)	7 (10.0)	
Smoking status at recruitment				0.438†
Never smoked	45 (72.6)	51 (71.8)	51/69 (73.9)	
Smoker before pregnancy	13 (21.0)	19 (26.8)	17/69 (24.6)	
Smoker before and during pregnancy	4 (6.5)	1 (1.4)	1/69 (1.4)	
Low educational level	15/61 (24.6)	11/70 (15.7)	9/69 (13.0)	0.203‡
Unemployed	9/61 (14.8)	14/70 (20.0)	12/69 (17.4)	0.754‡
Pre‐existing medical condition				
Hypertension	0 (0)	1/70 (1.4)	2/68 (2.9)	0.649†
Autoimmune disease	4 (6.5)	4 (5.6)	4 (5.7)	> 0.999†
Type‐1 DM	0 (0)	0 (0)	3 (4.3)	0.067†
Type‐2 DM	0 (0)	0 (0)	0 (0)	NA
Hypothyroidism	6 (9.7)	9 (12.7)	11 (15.7)	0.585‡
Mode of conception				0.630†
Spontaneous	49 (79.0)	55 (77.5)	55 (78.6)	
IVF	10 (16.1)	14 (19.7)	8 (11.4)	
Egg donation	2 (3.2)	1 (1.4)	4 (5.7)	
Artificial insemination	1 (1.6)	1 (1.4)	3 (4.3)	
Risk of PE at first‐trimester screening				0.298‡
Low risk	47 (75.8)	52 (73.2)	52 (74.3)	
High risk	7 (11.3)	12 (16.9)	15 (21.4)	
No screening	8 (12.9)	7 (9.9)	3 (4.3)	
Aspirin treatment	9 (14.5)	11 (15.5)	17 (24.3)	0.267‡
LMWH treatment	4 (6.5)	4 (5.6)	5 (7.1)	0.938†

Data are given as mean ± SD, *n* (%) or *n*/*N* (%). *P*‐values were calculated using:

* one‐way ANOVA,

† Fisher–Freeman–Halton exact test or

‡ chi‐square test. BMI, body mass index; DM, diabetes mellitus; GA, gestational age; IVF, *in‐vitro* fertilization; LMWH, low‐molecular‐ weight heparin; NA, not applicable; PE, pre‐eclampsia.

Perinatal outcomes are summarized in Table 2. The prevalence of gestational hypertension was significantly higher among women in the highest tertile of sFlt‐1/PlGF ratio (*P* < 0.001). There was a non‐significant trend towards an increased prevalence of pre‐eclampsia and fetal growth restriction from the lowest to the highest tertile. Gestational age at delivery decreased progressively across sFlt‐1/PlGF ratio tertiles, from a mean ± SD of 40 + 1 ± 0 + 6 weeks in the lowest tertile to 38 + 6 ± 1 + 0 weeks in the highest tertile (*P* < 0.001).

**Table 2:** Perinatal outcome in 203 women, according to tertile of soluble fms‐like tyrosine kinase‐1 to placental growth factor (sFlt‐1/PlGF) ratio

	sFlt‐1/PlGF ratio tertile			
Outcome	Lowest (*n* = 62)	Middle (*n* = 71)	Highest (*n* = 70)	*P*
Gestational hypertension	0 (0)	2 (2.8)	18 (25.7)	< 0.001*
Pre‐eclampsia	0 (0)	1 (1.4)	4 (5.7)	0.107†
Gestational diabetes	2 (3.2)	4 (5.6)	4 (5.7)	0.780†
Small‐for‐gestational age	0 (0)	2 (2.8)	2/69 (2.9)	0.551†
Fetal growth restriction	0 (0)	1 (1.4)	4/69 (5.8)	0.107†
Fetal macrosomia	5 (8.1)	5 (7.0)	6/69 (8.7)	0.948†
Labor onset				0.064†
Spontaneous	20 (32.3)	25 (35.2)	15/69 (21.7)	
Induction	41 (66.1)	42 (59.2)	45/69 (65.2)	
Elective CS	1 (1.6)	4 (5.6)	9/69 (13.0)	
Type of delivery				0.114†
Vaginal delivery	42 (67.7)	53 (74.6)	39/69 (56.5)	
Instrumental delivery	5 (8.1)	2 (2.8)	3/69 (4.3)	
CS	15 (24.2)	16 (22.5)	27/69 (39.1)	
Emergency CS for fetal distress	3/15 (20.0)	5/16 (31.3)	5/27 (18.5)	0.662†
Gestational age at delivery (weeks)	40 + 1 ± 0 + 6	39 + 6 ± 1 + 0	38 + 6 ± 1 + 0	< 0.001‡
Birth weight < 3^rd^ centile	3 (4.8)	2 (2.8)	3/69 (4.3)	0.820†
Birth weight 3^rd^–10^th^ centile	4 (6.5)	7 (9.9)	8/69 (11.6)	0.596*
Birth weight > 90^th^ centile	10 (16.1)	8 (11.3)	5/69 (7.2)	0.276*
Female sex	30 (48.4)	33 (46.5)	35/69 (50.7)	0.907*
5‐min Apgar score < 7	0/60 (0)	0/70 (0)	1/68 (1.5)	0.646†
Neonatal acidosis	4/53 (7.5)	3/56 (5.4)	4/52 (7.7)	0.859†
Admission to NICU	2/61 (3.3)	2/69 (2.9)	1/66 (1.5)	0.866†

Data are given as *n* (%), *n*/*N* (%) or mean ± SD. *P*‐values were calculated using:

* chi‐square test,

† Fisher–Freeman–Halton exact test or

‡ one‐way ANOVA. CS, Cesarean section; NICU, neonatal intensive care unit.

**Figure 1:**
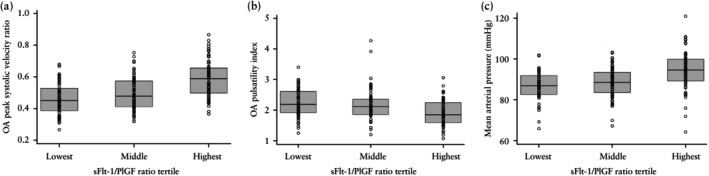
Box plots showing distribution of ophthalmic artery (OA) peak systolic velocity ratio (a), OA pulsatility index (b) and mean arterial pressure (c) in 203 women, according to tertile of soluble fms‐like tyrosine kinase‐1 to placental growth factor (sFlt‐1/PlGF) ratio. Boxes with internal lines represent median and interquartile range.

The results of the primary analysis are presented in Table 3 and Figure 1. With increasing sFlt‐1/PlGF ratio tertile, the OA‐PSV ratio increased significantly (median, 0.45 (interquartile range (IQR), 0.39–0.53) *vs* 0.48 (IQR, 0.41–0.58) *vs* 0.59 (IQR, 0.50–0.66); *P <* 0.001) and OA‐PI decreased significantly (median, 2.20 (IQR, 1.92–2.61) *vs* 2.13 (IQR, 1.86–2.37) *vs* 1.86 (IQR, 1.60–2.26); *P* = 0.002). Both associations remained statistically significant after adjustment for confounding variables, including maternal BMI, age and smoking status (adjusted *P* < 0.001 and *P* = 0.031, respectively). MAP also showed a significant upward trend with increasing sFlt‐1/PlGF ratio tertile (median, 87.0 (IQR, 82.7–92.0) mmHg *vs* 88.7 (IQR, 83.7–93.7) mmHg *vs* 94.7 (IQR, 89.3–100.0) mmHg; *P* < 0.001; adjusted *P* < 0.001). Although OA‐PSV2 was associated significantly with sFlt‐1/PlGF ratio tertile in the unadjusted analysis (*P* = 0.038), this association did not remain significant after adjustment (adjusted *P* = 0.149). No significant trend was observed in mean UtA‐PI across sFlt‐1/PlGR ratio tertiles (*P* = 0.236). Table S2 presents the sensitivity analysis restricted to the cases that met the quality criteria for OA Doppler assessment (*n* = 189). The associations of the OA‐PSV ratio, OA‐PI and MAP with sFlt‐1/PlGF ratio tertile remained statistically significant on both unadjusted and adjusted analysis.

**Table 3:** Associations of ophthalmic artery (OA) and maternal–fetal Doppler indices with soluble fms‐like tyrosine kinase‐1 to placental growth factor (sFlt‐1/PlGF) ratio in 203 women

	sFlt‐1/PlGF ratio tertile				
Parameter	Lowest (*n* = 62)	Middle (*n* = 71)	Highest (*n* = 70)	*P*_trend_ *	Adjusted *P*_trend_ *, †
OA‐PSV1 (cm/s)	39.3 (34.7–43.5)	36.1 (31.5–42.9)	37.6 (32.2–42.7)	0.507	0.010
OA‐PSV2 (cm/s)	18.7 (12.9–21.7)	17.4 (13.1–22.8)	22.0 (17.6–25.9)‡, §	0.038	0.149
OA‐PSV ratio	0.45 (0.39–0.53)	0.48 (0.41–0.58)	0.59 (0.50–0.66)‡, §	< 0.001	< 0.001
OA‐PI	2.20 (1.92–2.61)	2.13 (1.86–2.37)	1.86 (1.60–2.26)‡, §	0.002	0.031
EFW (g)	2949 (2817–3165)	2923 (2760–3143)	2924 (2678–3172)	0.828	0.171
UA‐PI	0.87 (0.77–0.95)	0.86 (0.75–0.94)	0.86 (0.77–0.97)	0.990	0.916
MCA‐PI	1.60 (1.45–1.83)	1.59 (1.46–1.85)	1.60 (1.41–1.82)	0.990	0.878
CPR	1.94 (1.64–2.26)	1.85 (1.69–2.21)	1.86 (1.53–2.19)	0.377	0.196
Mean UtA‐PI	0.64 (0.55–0.73)	0.65 (0.58–0.77)	0.67 (0.54–0.82)	0.236	0.213
MAP (mmHg)	87.0 (82.7–92.0)	88.7 (83.7–93.7)	94.7 (89.3–100.0)‡, §	< 0.001	< 0.001

Data are given as median (interquartile range).

* Jonckheere–Terpstra test.

† Adjusted for maternal body mass index, age and smoking status. Statistically significant compared with lowest tertile

‡ and with middle tertile

§ , by quantile regression. CPR, cerebroplacental ratio; EFW, estimated fetal weight; MAP, mean arterial pressure; MCA, middle cerebral artery; PI, pulsatility index; PSV1/2, first/second peak systolic velocity; UA, umbilical artery; UtA, uterine artery.

**Figure 2:**
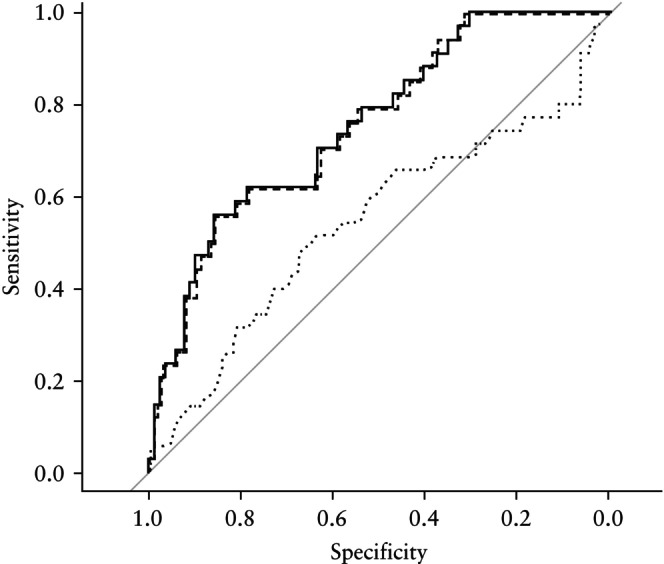
Receiver‐operating‐characteristics curves for prediction of soluble fms‐like tyrosine kinase‐1 to placental growth factor ratio ≥ 38 by ophthalmic artery peak systolic velocity (OA‐PSV) ratio (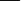), mean uterine artery pulsatility index (UtA‐PI) (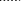) and combined model including OA‐PSV ratio and mean UtA‐PI (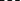).

The secondary exploratory analysis is summarized in Table 4 and Figure 2. A sFlt‐1/PlGF ratio of ≥ 38 was recorded in 35/203 (17.2%) participants. The OA‐PSV ratio demonstrated greater discriminative capacity compared with mean UtA‐PI (area under the ROC curve, 0.75 (95% CI, 0.67–0.84) *vs* 0.54 (95% CI, 0.42–0.66); *P* = 0.008). An OA‐PSV ratio of < 0.61 had positive and negative predictive values for a sFlt‐1/PlGF ratio ≥ 38 of 42.8% (95% CI, 32.1–48.6%) and 90.5% (95% CI, 86.7–93.5%), respectively, at a 15% false‐positive rate. Combining the OA‐PSV ratio with mean UtA‐PI did not improve discriminative performance.

**Table 4:** Predictive performance of Doppler models for soluble fms‐like tyrosine kinase‐1 to placental growth factor (sFlt‐1/PlGF) ratio ≥ 38

Model	DR (%)	PPV (%)	NPV (%)	AUC
OA‐PSV ratio				0.75 (0.67–0.84)
5% FPR	23.5 (8.8–44.1)	48.6 (26.2–64.0)	86.0 (83.8–89.4)	
10% FPR	41.1 (17.7–61.8)	45.3 (26.2–55.4)	88.4 (84.5–92.1)	
15% FPR	55.9 (32.4–70.6)	42.8 (32.1–48.6)	90.5 (86.7–93.5)	
Mean UtA‐PI				0.54 (0.42–0.66)
5% FPR	6.8 (0.0–21.1)	22.2 (0.0–46.4)	83.0 (82.0–85.2)	
10% FPR	14.3 (2.9–28.6)	22.9 (5.6–37.1)	83.4 (81.6–85.8)	
15% FPR	19.4 (6.1–40.0)	21.3 (8.1–35.7)	83.5 (81.3–87.2)	
OA‐PSV ratio + mean UtA‐PI				0.75 (0.67–0.84)
5% FPR	23.5 (11.8–44.2)	49.1 (26.6–64.4)	85.9 (83.6–89.3)	
10% FPR	41.1 (17.7–61.8)	45.8 (26.6–55.9)	88.2 (84.2–92.0)	
15% FPR	52.9 (32.4–70.6)	42.0 (30.6–49.1)	89.8 (86.0–93.4)	

Values in parentheses are 95% CI. Detection rate (DR), positive predictive value (PPV) and negative predictive value (NPV) reflect ability of each Doppler parameter to discriminate sFlt‐1/PlGF ratio ≥ 38. Values are presented at predefined false‐positive rates (FPR) of 5%, 10% and 15%. AUC, area under receiver‐operating‐characteristics curve; OA‐PSV, ophthalmic artery peak systolic velocity; UtA‐PI, uterine artery pulsatility index.

## DISCUSSION

### Main findings

This study provides new evidence of a significant association between the sFlt‐1/PlGF ratio and OA Doppler parameters in near‐term pregnancies. Specifically, a higher sFlt‐1/PlGF ratio was associated with an increase in the OA‐PSV ratio and a decrease in OA‐PI, even without clinical suspicion of pre‐eclampsia. In contrast, no significant relationship was observed between UtA‐PI and angiogenic status at near term. Although this study was not designed to assess diagnostic performance, our exploratory analysis suggested that the OA‐PSV ratio could be a promising tool for ruling out a sFlt‐1/PlGF ratio ≥ 38. The inclusion of UtA Doppler in our combined model did not improve the predictive performance for a sFlt‐1/PlGF ratio ≥ 38.

### Comparison with previous studies

Robust evidence indicates that individuals with pre‐eclampsia exhibit hemodynamic changes in OA Doppler indices, particularly an increased PSV ratio and a reduced PI^17^, ^18^, ^26^, ^39^, ^40^, ^41^. Among these, the OA‐PSV ratio has shown the most consistent performance, especially for predicting late‐onset pre‐eclampsia^19^. Given these promising results, recent studies have explored the integration of OA Doppler into multivariable prediction models^20^, ^32^. Lau *et al*.^32^ found that combining the OA‐PSV ratio with maternal factors and MAP improved the prediction of imminent pre‐eclampsia compared with angiogenic markers alone, suggesting that the OA‐PSV ratio could potentially replace the sFlt‐1/PlGF ratio. While this proposition is appealing, particularly for settings with limited access to biochemical testing, it is crucial to distinguish between prediction and association. A variable can predict an outcome without being involved directly in its underlying physiological mechanisms.

To our knowledge, this is the first study to assess formally the association between OA Doppler parameters and the sFlt‐1/PlGF ratio in near‐term pregnancies. However, previous studies indirectly support this relationship. Lau *et al*.^28^ reported that the OA‐PSV ratio distinguishes pre‐eclampsia from other hypertensive disorders, similar to angiogenic markers^42^. Furthermore, the OA‐PSV ratio was reported to be significantly higher in individuals with severe pre‐eclampsia compared to those with milder forms^43^. An OA‐PSV ratio cut‐off of > 0.78 demonstrated strong discriminatory ability for severe pre‐eclampsia^44^, while values > 0.99 have been associated with adverse maternal outcome^45^. These findings parallel those of angiogenic markers, which not only distinguish pre‐eclampsia from other hypertensive disorders, but also indicate the severity and prognosis of the disease^14^.

Further supporting our findings, there is evidence to suggest that normotensive individuals with a small‐for‐gestational‐age or growth‐restricted fetus show higher OA‐PSV ratios and lower OA‐PI values^46^, ^47^, ^48^. Likewise, normotensive individuals with Type‐1 diabetes mellitus exhibit reduced OA‐PI^49^. The link between these conditions and endothelial dysfunction reinforces our hypothesis that OA Doppler may reflect systemic vascular alterations consistent with angiogenic imbalance, independently of blood pressure.

Finally, consistent with previous work^30^, we found no significant association between UtA‐PI and angiogenic imbalance. This lends support to a growing body of evidence suggesting that pre‐eclampsia at term is due primarily to maternal cardiovascular maladaptation rather than placental insufficiency^6^.

### Pathophysiological mechanisms

Increased sFlt‐1 and decreased PlGF represent the biochemical signature of endothelial dysfunction in pre‐eclampsia, leading to systemic vasoconstriction, increased systemic vascular resistance and impaired vascular autoregulation^11^, ^22^. According to hemodynamic models, these alterations are reflected in the Doppler waveform of the OA, which is characterized by two distinct systolic peaks. The first (P1) corresponds to ventricular ejection, while the second (P2) arises from the pulse wave rebounding from the peripheral vascular bed. In this context, elevated systemic vascular resistance enhances the reflected wave, increasing the P2/P1 ratio^26^, ^50^. Therefore, OA Doppler may not only indicate vascular resistance^27^ but it may also serve as a functional marker of endothelial damage caused by angiogenic imbalance.

Our findings suggest that OA Doppler may also capture cerebrovascular alterations associated with angiogenic imbalance. In 1994, Hata *et al*.^24^ first reported a decrease in OA‐PI in individuals with pre‐eclampsia, suggesting a state of cerebral vasodilation rather than vasoconstriction^24^, ^25^. This paradoxical hyperperfusion is thought to result from impaired cerebral autoregulation^51^ secondary to endothelial disruption at the blood–brain barrier, mediated by circulating antiangiogenic factors^52^, ^53^. The consequent increase in vascular permeability facilitates plasma extravasation and vasogenic edema^54^, which are hallmarks of severe neurological complications of pre‐eclampsia, including posterior reversible encephalopathy syndrome, which has also been observed in patients undergoing antiangiogenic therapy.

### Clinical and research implications

Establishing relationships between OA Doppler parameters and angiogenic markers is essential to determine whether they reflect shared or distinct physiological mechanisms. The clinical relevance of our findings lies in the potential of OA Doppler as a low‐cost, accessible, non‐invasive surrogate for angiogenic markers, which is especially pertinent for settings in which biochemical testing is limited. Integrating OA Doppler into third‐trimester assessment could enable the early detection of subclinical angiogenic imbalance, targeted surveillance and optimized timing of delivery. Our study also fills a knowledge gap by demonstrating this association at near term and supporting future research to define OA Doppler cut‐offs that accurately reflect angiogenic imbalance.

### Strengths and limitations

A major strength of this study is its prospective design, with Doppler assessments performed by a single trained operator blinded to angiogenic results, ensuring consistency and minimizing measurement bias. Although this limited the evaluation of interobserver variability, it supported the internal validity of the study. Nevertheless, several limitations should be acknowledged. The cross‐sectional design and relatively small sample size restrict causal inference and full assessment of the predictive performance of Doppler indices. Given the exploratory nature of the analyses, our findings should be interpreted with caution. Second, restriction of the study population to nulliparous participants limits the generalizability of our findings. Additionally, we were unable to assess the incidence of pre‐eclampsia and the predictive capability of OA Doppler for pre‐eclampsia, as the cohort was part of the PE37 trial, in which 80% of individuals with a sFlt‐1/PlGF ratio > 90^th^ centile underwent induction of labor. Finally, while it is tempting to consider OA Doppler as a surrogate for angiogenic factors, our findings are limited to its use as a rule‐out tool, and larger studies are needed to evaluate other cut‐off values.

### Conclusions

This study demonstrates a significant association between OA Doppler parameters and the sFlt‐1/PlGF ratio in near‐term pregnancies. Our findings suggest that OA Doppler indices, particularly the PSV ratio, reflect angiogenic imbalance. Given its non‐invasive nature, accessibility and low cost, OA Doppler emerges as a promising surrogate tool for ruling out angiogenic imbalance. Further studies are needed to validate this finding and explore its clinical utility.

## Supporting information


**Table S1** Tertile cut‐offs for soluble fms‐like tyrosine kinase‐1 to placental growth factor ratio obtained from external sample of 654 women by quantile regression.

**Table S2** Sensitivity analysis of associations of ophthalmic artery and maternal–fetal Doppler indices with soluble fms‐like tyrosine kinase‐1 to placental growth factor (sFlt‐1/PlGF) ratio in 189 women meeting quality criteria for ophthalmic artery Doppler evaluation.

## Data Availability

The data that support the findings of this study are available on request from the corresponding author. The data are not publicly available due to privacy or ethical restrictions.
