# Supplementary material for: Ophthalmic artery Doppler as potential surrogate marker of angiogenic imbalance in near‐term pregnancy
Source: Ultrasound Obstet Gynecol. 2026 Jun 28;68(2):211–9. doi: 10.1002/uog.70270 (PMC13432983; doi:10.1002/uog.70270)
Supplement: Supplementary file 1 — Table S1 Tertile cut‐offs for soluble fms‐like tyrosine kinase‐1 to placental growth factor ratio obtained from external sample of 654 women by quantile regression. [file UOG-68-211-s002.docx]

**Table S1** Tertile cut-offs for soluble fms-like tyrosine kinase-1 to placental growth factor ratio obtained from external sample of 654 women by quantile regression.

| **sFlt-1/PlGF ratio** | **1^st^ tertile** | **3^rd^ tertile** |
| --- | --- | --- |
|  | t1-34.833+ GA[weeks]*1.167 | t3=-138.25+ GA[weeks]*4.375 |
| 35^+0^ | 6.0 | 14.9 |
| 35^+1^ | 6.2 | 15.5 |
| 35^+2^ | 6.3 | 16.1 |
| 35^+3^ | 6.5 | 16.8 |
| 35^+4^ | 6.7 | 17.4 |
| 35^+5^ | 6.8 | 18.0 |
| 35^+6^ | 7.0 | 18.6 |
| 36^+0^ | 7.2 | 19.3 |
| 36^+1^ | 7.3 | 19.9 |
| 36^+2^ | 7.5 | 20.5 |
| 36^+3^ | 7.7 | 21.1 |
| 36^+4^ | 7.8 | 21.8 |
| 36^+5^ | 8.0 | 22.4 |
| 36^+6^ | 8.2 | 23.0 |
| 37^+0^ | 7.2 | 19.3 |
| 37^+1^ | 8.5 | 24.3 |
| 37^+2^ | 8.7 | 24.9 |
| 37^+3^ | 8.8 | 25.5 |
| 37^+4^ | 9.0 | 26.1 |
| 37^+5^ | 9.2 | 26.8 |
| 37^+6^ | 9.3 | 27.4 |

GA: gestational age.
