# Supplementary material for: Ophthalmic artery Doppler as potential surrogate marker of angiogenic imbalance in near‐term pregnancy
Source: Ultrasound Obstet Gynecol. 2026 Jun 28;68(2):211–9. doi: 10.1002/uog.70270 (PMC13432983; doi:10.1002/uog.70270)
Supplement: Supplementary file 2 — Table S2 Sensitivity analysis of associations of ophthalmic artery and maternal–fetal Doppler indices with soluble fms‐like tyrosine kinase‐1 to placental growth factor (sFlt‐1/PlGF) ratio in 189 women meeting quality criteria for ophthalmic artery Doppler evaluation. [file UOG-68-211-s001.docx]

**Table S2** Sensitivity analysis of associations of ophthalmic artery and maternal–fetal Doppler indices with soluble fms-like tyrosine kinase-1 to placental growth factor (sFlt-1/PlGF) ratio in 189 women meeting quality criteria for ophthalmic artery Doppler evaluation

| **Parameter** | **sFlt-1/PIGF ratio tertile** | | | ***P*_trend_^+^** | **Adjusted *P*_trend_*** |
| --- | --- | --- | --- | --- | --- |
|  | **Lowest (*n* = 60)** | **Middle (*n* = 64)** | **Highest (*n* = 65)** |  |  |
| OA-PSV1 | 39.7 (35.1–43.5) | 36.6 (31.6–42.8) | 37.6 (33.2–43.6) | 0.446 | 0.026 |
| OA-PSV2 | 18.9 (13.0-21.7) | 17.8 (13.5-23.1) | 22.9 (18.5-26.3)†‡ | 0.006 | 0.092 |
| OA-PSV ratio | 0.45 (0.39–0.52) | 0.47 (0.41–0.57) | 0.59 (0.51–0.66) †‡ | < 0.001 | < 0.001 |
| OA-PI | 2.1 (1.93–2.62) | 2.12 (1.86–2.36) | 1.85 (1.58–2.26) †‡ | 0.001 | 0.023 |
| EFW | 2956 (2825–3166) | 2914 (2749–3139) | 2966 (2750–3172) | 0.924 | 0.295 |
| UA-PI | 0.87 (0.78–0.95) | 0.87 (0.74–0.95) | 0.87 (0.77–0.97) | 0.990 | 0.609 |
| MCA-PI | 1.60 (1.45–1.84) | 1.59 (1.46–1.86) | 1.60 (1.41–1.82) | 0.990 | 0.961 |
| CPR | 1.92 (1.64–2.23) | 1.86 (1.67–2.24) | 1.86 (1.53–2.17) | 0.591 | 0.199 |
| Mean UtA-PI | 0.64 (0.55–0.72) | 0.65 (0.58–0.76) | 0.67 (0.55–0.83) | 0.078 | 0.215 |
| MAP | 87.3 (82.8–92.2) | 88.5 (85.0–93.7) | 94.7 (88.7–100.0) †‡ | < 0.001 | < 0.001 |

Data are given as median (interquartile range). + Jonckheere–Terpstra test; *Adjusted for maternal body mass index, age and smoking status. Statistically significant compared with lowest tertile† and with middle tertile‡, by quantile regression. CPR, cerebroplacental ratio; EFW, estimated fetal weight; MAP, mean arterial pressure; MCA, middle cerebral artery; OA, ophthalmic artery; PI, pulsatility index; PSV, peak systolic velocity; UA, umbilical artery; UtA, uterine artery.
